# A factor score reflecting cognitive functioning in patients from the Swiss Atrial Fibrillation Cohort Study (Swiss-AF)

**DOI:** 10.1371/journal.pone.0240167

**Published:** 2020-10-09

**Authors:** Anne Springer, Andreas U. Monsch, Gilles Dutilh, Michael Coslovsky, Rogier A. Kievit, Leo H. Bonati, David Conen, Stefanie Aeschbacher, Juerg H. Beer, Matthias Schwenkglenks, Urs Fischer, Christine S. Meyer-Zuern, Giulio Conte, Elisavet Moutzouri, Giorgio Moschovitis, Michael Kühne, Stefan Osswald, on behalf of the Swiss-AF Study Investigators

**Affiliations:** 1 Cardiology Division, Department of Medicine, University Hospital Basel, Basel, Switzerland; 2 Cardiovascular Research Institute Basel (CRIB), Basel, Switzerland; 3 Memory Clinic, University Department of Geriatric Medicine FELIX PLATTER, Basel, Switzerland; 4 Faculty of Psychology, University of Basel, Basel, Switzerland; 5 Department of Clinical Research, Clinical Trial Unit, University of Basel Hospital, Basel, Switzerland; 6 Donders Institute for Brain, Cognition and Behaviour, Radboud University, Nijmegen, Netherlands; 7 Department of Neurology and Stroke Center, University Hospital Basel, University of Basel, Basel, Switzerland; 8 Population Health Research Institute, McMaster University, Hamilton, Canada; 9 Department of Medicine, Cantonal Hospital of Baden and Molecular Cardiology, University Hospital of Zurich, Zurich, Switzerland; 10 Epidemiology, Biostatistics, and Prevention Institute, University of Zurich, Zurich, Switzerland; 11 Department of Neurology, Inselspital, Bern University Hospital, University of Bern, Switzerland; 12 Cardiocentro Ticino, Lugano, Switzerland; 13 Institute of Primary Health Care (BIHAM), University of Bern, Bern, Switzerland; 14 Department of General Internal Medicine, Inselspital Bern, University Hospital, University of Bern, Bern, Switzerland; 15 Division of Cardiology, Ospedale Regionale di Lugano EOC, Lugano, Switzerland; Universidade de Sao Paulo, BRAZIL

## Abstract

**Background:**

Atrial fibrillation (AF), the most common sustained cardiac arrhythmia, is considered as risk factor for the development of mild cognitive impairment (MCI) and dementia. However, dynamics of cognitive functions are subtle, and neurocognitive assessments largely differ in detecting these changes. We aimed to develop and evaluate a score which represents the common aspects of the cognitive functions measured by validated tests (i.e., “general cognitive construct”), while reducing overlap between tests and be more sensitive to identify changes in overall cognitive functioning.

**Methods:**

We developed the CoCo (cognitive construct) score to reflect the cognitive performance obtained by all items of four neurocognitive assessments (Montreal Cognitive Assessment (MoCA); Trail Making Test; Semantic Fluency, animals; Digital Symbol Substitution Test). The sample comprised 2,415 AF patients from the Swiss Atrial Fibrillation Cohort Study (Swiss-AF), 87% aged at least 65 years. Psychometric statistics were calculated for two cognitive measures based on (i) the full set of items from the neurocognitive test battery administered in the Swiss-AF study (i.e., CoCo item set) and (ii) the items from the widely used MoCA test. For the CoCo item set, a factor score was derived based on a principal component analysis, and its measurement properties were analyzed.

**Results:**

Both the MoCA item set and the full neurocognitive test battery revealed good psychometric properties, especially the full battery. A one-factor model with good model fit and performance across time and groups was identified and used to generate the CoCo score, reflecting for each patient the common cognitive skill performance measured across the full neurocognitive test battery. The CoCo score showed larger effect sizes compared to the MoCA score in relation to relevant clinical variables.

**Conclusion:**

The derived factor score allows summarizing AF patients’ cognitive performance as a single score. Using this score in the Swiss-AF project increases measurement sensitivity and decreases the number of statistical tests needed, which will be helpful in future studies addressing how AF affects the risk of developing cognitive impairment.

## Introduction

The global number of people living with dementia is growing rapidly, constituting a major challenge to health-care systems, working societies, and families worldwide. Atrial fibrillation (AF), the most common sustained cardiac arrhythmia, is considered a key risk condition for the development of dementia, alongside age and other risk factors [1–5]. It has recently been reported that patients with AF aged 65 years or older have a relatively high burden of brain lesions, including overt and clinically unrecognized vascular brain lesions, micro-bleeds, and other structural brain lesions [6]. Furthermore, these brain lesions were associated with reduced cognitive performance, even for AF patients with clinically silent infarcts [6–9]. These results can be linked to the concept of mild cognitive impairment (MCI), a syndrome that often precedes dementia [10, 11]. Specifically, MCI involves a measurable decline of cognitive functioning that does not fulfill the criteria of dementia, because basic functional autonomy is not lost [12, 13]. According to the 5th edition of the Diagnostic and Statistical Manual of Mental Disorders (DSM-5) [14], the etiologies of mild neurocognitive disorders–which is a newer term for MCI–are manifold. The most common cause is Alzheimer's disease, but depending on specific clinical features, course, and pattern of cognitive impairment, other etiologies must be considered. For example, the term “mild vascular neurocognitive disorder” refers to a MCI where clinical features are consistent with a vascular etiology, as suggested by either the temporal relationship of cognitive deficits and cerebrovascular events and/or evidence of decline that is prominent in complex attention (including processing speed) and frontal executive function [14].

To date, the mechanisms in AF patients underlying the development of MCI preceding dementia–or major neurocognitive disorders in the APA [14] nomenclature–remain poorly understood. The Swiss Atrial Fibrillation Cohort Study (Swiss-AF) offers a unique opportunity to address this issue. It provides a large, well-described sample of patients with AF, who undergo neurocognitive assessment at annual follow-up visits. This allows for investigations of basic cognitive functions, including attention, psychomotor speed, and mental flexibility (executive control) as well as short-term memory, language, and visuo-spatial abilities. In general, these cognitive functions enable individuals to plan, to remember, and to focus and shift their attention, thus ensuring appropriate and goal-directed behavior in constantly changing situations.

The main goal of the current study was to create a single score that reflects the performance measured by all items of the different neuropsychological tests used in the Swiss-AF cohort through their common aspects. We refer to this score as CoCo (cognitive construct) score. Specifically, in contrast to a *composite score*, we aimed at a *factor score* expressing a latent factor, corresponding to a reflective model [15].

Furthermore, we aimed to analyze the psychometric properties of the new score and to optimize its measurement properties. Aggregating the scores obtained by the different neuropsychological tests used into one single reliable score (the CoCo score) will reduce the number of statistical tests that would be needed when analyzing each of these test scores separately. Moreover, the intended score is likely to be more granular and more sensitive to detect small changes in cognitive function, which may be missed when examining each neurocognitive test alone [16]. Hence, in future studies of the Swiss-AF population, we may use the new CoCo score to identify changes in cognitive function and to study their associations with crucial risk factors (e.g., brain lesions, clinical variables, lifestyle parameters).

## Materials and methods

### Study design and patient population

This was a cross-sectional analysis using baseline data from the Swiss-AF cohort study (NCT02105844), which has been described in detail elsewhere [6, 17]. The leading Ethics Committee–Ethikkommission Nordwest- und Zentralschweiz (EKNZ)–fully approved the study procedures (Approval No PB_2016_00793). Each patient signed a written informed consent.

Briefly, Swiss-AF is an ongoing prospective, observational cohort study that included 2,415 patients (662 (27.4%) women; mean age ± standard deviation (SD) 73.24 ± 8.4), enrolled between 2014 and 2017 across 14 centers in Switzerland. Patients were enrolled if they were at least 65 years old (in addition, 200 patients aged between 45–65 years were enrolled to assess socio-economic aspects of AF in the working population). 87% of patients included in the present analysis were 65 years or older (n = 2,100).

Eligible patients had to have a history of documented AF [17], i.e., paroxysmal AF (at least twice within the last 60 months), persistent AF (documented within the last 60 months by ECG or rhythm monitoring devices) or permanent AF, as defined according to the guidelines of the European Society of Cardiology [18].

Patients were excluded if they indicated only secondary, reversible episodes of AF (e.g., after cardiac surgery or severe sepsis), any acute illness within the last 4 weeks (while being eligible after stabilization of the acute episode), or if they were unable to understand, to date and to sign the patient informed consent form (e.g., patients with dementia, psychosis or delirium).

No further requirements regarding the integrity of cognitive abilities were defined since we aimed to establish a representative large sample of elderly patients with diagnosed AF. However, we safeguarded against individuals with overt dementia, as those who were unable to give informed consent for their participation and those unable to attend the 1.5–2 hours`baseline study visit at the hospital, including brain MRI investigation, ECG and clinical measurements as well as extensive case report forms (CRFs) with neurocognitive assessment, were not included in the study.

Eligible patients were found by screening in- and outpatients of the participating hospitals and by contacting general practitioners in the area.

### Clinical measures and brain magnetic resonance imaging (bMRI)

Information on personal characteristics, risk factors, co-morbidities, current medication, medication adherence, and other variables (e.g., weight, height, blood pressure) were collected using standardized CRFs [17].

A standardized bMRI protocol that does not require application of contrast agents was installed on an MR scanner at each participating site, at either 1.5 or 3 Tesla [17]. Individual patients were investigated on the same scanner with identical sequence protocol, at baseline (and after 2 years, if possible). All images were centrally analyzed by trained MRI technicians and validated by board-certified radiologists according to a pre-specified analysis plan; lesions were evaluated using the AMIRA software which calculates volumes and number of lesions [17].

### Neurocognitive assessment

Cognitive functions were assessed with four validated, widely-used neurocognitive tests: The Montreal Cognitive Assessment (MoCA) [19], the Trail Making Test (TMT) [20], Semantic Fluency Test, animals (SFT) [21] and the Digit Symbol Substitution Test (DSST) [22]. All tests were administered in a paper-pencil format and, just as the CRFs, provided in the main national languages of Switzerland (i.e., German, French and Italian), which were administered depending on the patient's mother tongue (72,4% German vs. 11,9% French vs. 10,2% Italian, corresponding to the general language distribution in the Swiss population). As the majority of the patients included in this study were raised in exactly the same educational system in Switzerland, the test scores were not expected to substantially differ according to the language used. Furthermore, for the MoCA test, the official, validated versions for each of the three languages were used (www.mocatest.org), while both the TMT and the DSST are language- independent tests.

The *Montreal Cognitive Assessment (MoCA;* *www*.*mocatest*.*org**)* is a commonly used and validated screening tool for MCI, evaluating several cognitive domains including visuospatial abilities (e.g., clock drawing), memory, orientation, abstraction, and language. Furthermore, it measures different aspects of executive functions, i.e., mental flexibility (task shifting), attention, and working memory [19]. Patients can obtain a maximum of 30 points and a minimum of 0 points, with higher scores indicating better cognitive performance. For those who achieve less than 30 points and have equal or less than 12 years of education, one point is added to the MoCA total score.

The *Trail Making Test (TMT*) [20] is a common test of psychomotor speed and mental flexibility that is administered in two parts. In part A (TMT-A), the patient connects circled numbers in an ascending order (i.e., 1-2-3, etc.) by drawing a continuous line (trail) between them as quickly and as accurately as possible, enabling to measure visual attention and psychomotor speed. Part B (TMT-B) requires the subject to connect circled numbers and letters in an alternating numeric and alphabetic order (i.e., 1-A-2-B, etc.), again, with the same emphasis on speed and accuracy, assessing mental flexibility (i.e., task switching) [23, 24]. In order to account for different lengths of the trails A and B, we used the number of correct connections per second, i.e., speed, as dependent variable. That is, the test score was the time used divided by the total number of nodes correctly connected in that time. This measure has the advantage that it is valid for both, patients who finished the TMT in time and those who were not able to complete the TMT within the given maximum time, i.e., 180 seconds for TMT-A and 300 seconds for TMT-B.

The *Semantic Fluency Test*, *animals (SFT)* measures semantic memory, language production, and mental flexibility [21, 25, 26], complementing phonemic fluency within the MoCA test. Patients are asked to enumerate as many animal names as possible within 60 seconds. This requires to search for information from the semantic memory and to overtly produce the response. Previous evidence indicated that test performance (i.e., total number of correct words produced) depends on the size and location of brain lesions [27]. While semantic memory and word storage involve the temporal lobe, modulation of attention and word search depend on the frontal lobe [28] or the prefrontal-lateral cerebellar system [27].

Finally, the *Digit Symbol Substitution Test (DSST*) [22] allows to capture psychomotor speed, and performance is assumed to reflect the overall efficiency of cognitive operations [29, 30]. Patients receive a key grid of numbers and matching symbols (assigned to those numbers, respectively) and a test section with numbers and empty boxes. The task is to fill as many empty boxes as quickly as possible with the symbol that matches the corresponding number. A patient’s score is the number of correct number-symbol matches achieved within 120 seconds. We used this score as a continuous variable [31]. The DSST has a high test-retest reliability [32]. Previous evidence showed that psychomotor speed rapidly declines with older age [33], is associated with small vessel disease [34] and predictive of the onset of MCI and dementia [35–37].

Together, the neurocognitive test battery comprised 17 items. Table 1 provides a description of each item and its measurement level. We calculated the standard MoCA total score [19].

**Table 1:** Description of all 17 items included in the neurocognitive test battery administered in the Swiss-AF study. Items are grouped by test (MoCA, TrailMaking Test Part A and B (TMT-A, TMT-B), Semantic Fluency Test (SFT), and Digit Symbol Substitution Test, DSST) and indicated with definition of scores and measurement properties.

Item No	MoCA Items (scoring according to Manual; www.mocatest.org)
1	MoCA–Trail Making Test with letters and numbers; scored as "completed" vs "not completed": [0, 1]
2	Copy Cube; scored as "completed" vs "not completed": [1, 0]
3	Clock Drawing; scored as to how many of the three features are correct: [0, 1, 2, 3]
4	Naming Animals; scored as to the number of animals correctly named: [0, 1, 2, 3]
5	Digit Span forward; scored as "completed" vs "not completed": [1, 0]
6	Digit Span backward; scored as "completed" vs "not completed": [1, 0]
7	Letter A; scored as "completed" if less than 2 errors occurred: [1, 0]
8	100–7 (Serial 7 Subtraction); scored as: 0 correct [0 points], 1 through 3 correct [1 point], 4 correct [2 points], 5 correct [3 points]; values range from: [0, 1, 2, 3]
9	Sentence Repetition; scored according to number of sentences repeated correctly: [0, 1, 2]
10	F-Words, i.e., naming as many words that begin with the letter F; the number of correct words beginning with the letter F given in one minute [0,. . .] (scoring within the MoCA total = 11 or more points [1], ten or less [0])
11	Abstraction; scored as the number of correct similarities [0, 1, 2]
12	Delayed Recall; scored as the number of words correctly recalled [0, 1, 2, 3, 4, 5]
13	Orientation; scored as the number of correct answers: [0, 1, 2, 3, 4, 5]
	**Trail Making Test, Part A, Item**
14	Outcome: number of correct connections per second: [0,. . .]
	**Trail Making Test, Part B, Item**
15	Outcome: number of correct connections per second: [0,. . .]
	**Semantic Fluency, Animals (SF), Item**
16	Number of correct animal names given in one minute: [0,. . .]
	**Digit Symbol Substitution Test (DSST), Item**
17	Number of correct symbols filled out in 120 seconds: [0,. . .]

### Procedures and data quality

Data were collected during face-to-face on-site visits. At the beginning of the study, all study personnel underwent a standardized training of the study procedures before being qualified to evaluate patients and to enter the data into an electronic database. Specific emphasis was put on the neurocognitive assessment; a dedicated training video was created and made available for all investigators at all sites. New staff members were trained at site visits by experienced investigators from the University Hospital Basel, Switzerland. In addition, the Clinical Trial Unit of the University Hospital Basel provided regular reports of missing data that were transmitted to the sites for completion. Likewise, outliers of each variable were reported to the respective sites for cross-validation with source data, ensuring high data quality and completeness.

Of all 2,415 patients assessed at baseline, 57 patients had a missing value for at least one of the cognitive items. Most cases (82%) with a missing value only had one missing value, mostly due to the omission of the TMT. As our analyses were based on the correlation matrix of all items and pairwise complete cases, a missing value does not necessitate the omission of an entire patient’s data; thus, we performed no imputation.

### Statistical analyses

Statistical analyses were performed using *R* (Version 3.6.1) and the *Lavaan R* package for factor analysis [38]. First, the classic psychometric properties of the items in the full neurocognitive test battery were explored; i.e., we calculated Cronbach’s alpha and McDonald’s Omega providing information about the reliability of the corresponding cognitive measures. Then, we performed a principal components analyses (PCA) and draw a parallel plot to explore the dimensionality of the 17 items in the neurocognitive test battery. Based on the results of this PCA we performed an exploratory factor analysis (EFA) to explore one- and two-factor models to account for the data. The EFA was performed with varimax rotation using maximum likelihood estimation, and the models were fit to the polychoric correlation matrix, thereby taking account of the ordinal measurement level of some of the items in the neurocognitive test battery (see test item details in Table 1). In particular, we inspected which items would be assigned to which factor. As we will show, this exploration suggested that a one-factor model strikes the better balance between interpretability and model performance than a two-factor model. Because the thus selected one-factor model is intended to be applied to create scores on future follow-up measurements in the Swiss-AF cohort, group invariance and in particular, time-invariance of factor loadings is an essential property of this score. Therefore, we performed an analysis of both types of measurement invariance (MI). In this analysis, we included data of the baseline measurement as well as the first-follow up investigation. To test MI, we compared a model in which each item’s factor loading is constrained to be the same at baseline and follow-up to a model in which the factor loadings are free to vary between the two time-points. Similarly, we compared models where the factor loadings are constrained across a selection of relevant grouping variables. We then calculated the factor score for all patients at baseline. We show the applicability of this score by indicating how it relates to variables that are crucial in the Swiss-AF project: age, white matter lesions (WML), AF type, large non-cortical or cortical infarcts (LNCCIs) and small non-cortical infarcts (SNCIs) (Figs 2 and 3). In particular, the derived factor score showed a much clearer relation to crucial variables compared to the MoCA score (whose items are part of the derived factor score).

## Results

A total of 2,415 patients underwent cognitive assessments. Table 2 shows, for each item, the frequency and percentage of different scores (for binary (correct/incorrect) items and ordinally scored items), or the mean score and SDs (for continuously scored items) for each item from all neurocognitive tests. Table 2 also includes these summary statistics for the MoCA score and the CoCo score, as explained below. A correlation matrix of all items of the neurocognitive test battery is provided in S1 Table.

**Table 2:** Summary statistics for all items from all neurocognitive tests used, as well as for the MoCA score and the CoCo factor score. Statistics are shown for the complete analysis set.

Test Items	N (%) *or* mean (SD)
N	2,415
MoCA-Trail Making Test, N (%)	2,004 (83.2)
MoCA-Copy Cube, N (%)	1,591 (66.0)
MoCA-Clock Drawing, N (%)	
0	27 (1.1)
1	184 (7.6)
2	707 (29.3)
3	1,493 (61.9)
MoCA-Naming Animals, N (%)	
0	2 (0.1)
1	13 (0.5)
2	74 (3.1)
3	2,322 (96.3)
MoCA-Digit Span, forward, N (%)	2,114 (87.7)
MoCA-Digit Span, backward, N (%)	2,070 (85.8)
MoCA-Letter A, N(%)	2,258 (93.6)
MoCA-Serial 7 Subtraction, N (%)	
0	22 (0.9)
1	44 (1.8)
2	180 (7.5)
3	2,165 (89.8)
MoCA-Sentence Repetition, N (%)	
0	246 (10.2)
1	587 (24.3)
2	1,579 (65.5)
MoCA-F-Words, mean (SD)	9.70 (4.00)
MoCA-Abstraction, N (%)	
0	130 (5.4)
1	742 (30.8)
2	1,540 (63.8)
MoCA-Delayed Recall, mean (SD)	2.91 (1.63)
MoCA-Orientation, mean (SD)	5.91 (0.36)
TMT-A, connections per sec, mean (SD)	0.53 (0.21)
TMT-B, connections per sec, mean (SD)	0.21 (0.11)
Semantic Fluency, Animals, mean (SD)	18.88 (5.42)
Digit Symbol Substitution Test, mean (SD)	43.63 (14.27)
MoCAtotal (incl. +1 education), mean (SD)	25.35 (3.13)
CoCo score, mean (SD)	-0.00 (0.53)

### Psychometric properties

Basic psychometric properties were calculated for two item sets: 1) the CoCo item set, which comprises all items from the full neurocognitive test battery used in Swiss-AF (i.e., all items from MoCA, TMT-A and TMT-B, SFT, DSST) and (2) the MoCA test alone (i.e., the full MoCA test as described in Table 1).

We calculated Cronbach’s alpha and McDonald’s Omega for both item sets. While the results revealed that both indices are clearly reliable with values above .7, the CoCo items achieved higher internal consistency scores (Alpha = .84 and Omega = .86) as compared to the MoCA items (Alpha = .75 and Omega = .79).

For both item sets, we calculated further psychometric indices. The first two columns of Table 3 show, for each of the items, its correlation with an unweighted linear combination (sum) of all items and the correlation with the sum of all items except for the item itself. These correlations indicate how strong each item relates to the rest of the items. Note that for ordinal items, the reported correlations are polychoric correlations. The values in the third column show the reliability of the score when composed while excluding the relevant item. Values higher than the full score’s alpha would suggest potential improvement of the overall reliability of the unweighted cognitive score by removing the item. We find that the "alpha-if-item deleted" values for almost each item of the CoCo item set and for the MoCA item set are below the overall reliability score of both measures. The two rightmost columns show standardized factor loadings for each item for the CoCo score, as well as for the MoCA score, calculated by using "mean-variance adjusted weighted least-squares" (WLSMV) as optimization method.

**Table 3:** Overview of the psychometric item properties.

	r.cor	r.drop	std.alpha	CoCo
Trail Making Test, Part B	0.81	0.73	0.81	0.83
Digit Symbol Substitution Test	0.75	0.68	0.82	0.79
Trail Making Test, Part A	0.62	0.55	0.82	0.66
Semantic Fluency, Animals	0.61	0.56	0.82	0.61
MoCA-Trail Making Test	0.55	0.51	0.82	0.56
MoCA-Copy Cube	0.54	0.49	0.83	0.53
MoCA-Naming Animals	0.50	0.45	0.83	0.48
MoCA-Serial 7 Subtraction	0.52	0.48	0.83	0.47
MoCA-F-Words	0.45	0.41	0.83	0.45
MoCA-Abstraction	0.44	0.41	0.83	0.44
MoCA-Delayed Recall	0.44	0.40	0.83	0.44
MoCA-Sentence Repetition	0.43	0.39	0.83	0.38
MoCA-Clock Drawing	0.42	0.38	0.83	0.38
MoCA-Digit Span, forward	0.36	0.32	0.83	0.34
MoCA-Digit Span, backward	0.36	0.33	0.83	0.34
MoCA-Letter A	0.26	0.24	0.84	0.24
MoCA-Orientation	0.19	0.18	0.84	0.18

The first three columns show i) item-test correlations (correlation of each item with the sum of items), ii) item-rest correlations (correlation of each item with the sum of all items but itself), and iii) alpha-if-item deleted for each of the items of the full neurocognitive test battery (CoCo item set). The rightmost column shows standardized factor loadings for each item for the CoCo set (details of the factor score are provided in the section “Confirmatory factor analysis”).

The MoCA items "Letter A" and "Orientation" appear to be items that measure little in common with the rest of the test battery, indicated by the fact that the tests' alpha would increase slightly if these items were removed. Overall, these findings suggest that the 17 items of the neurocognitive test battery (the CoCo set) combined have a reasonable reliability.

**Fig 1:**
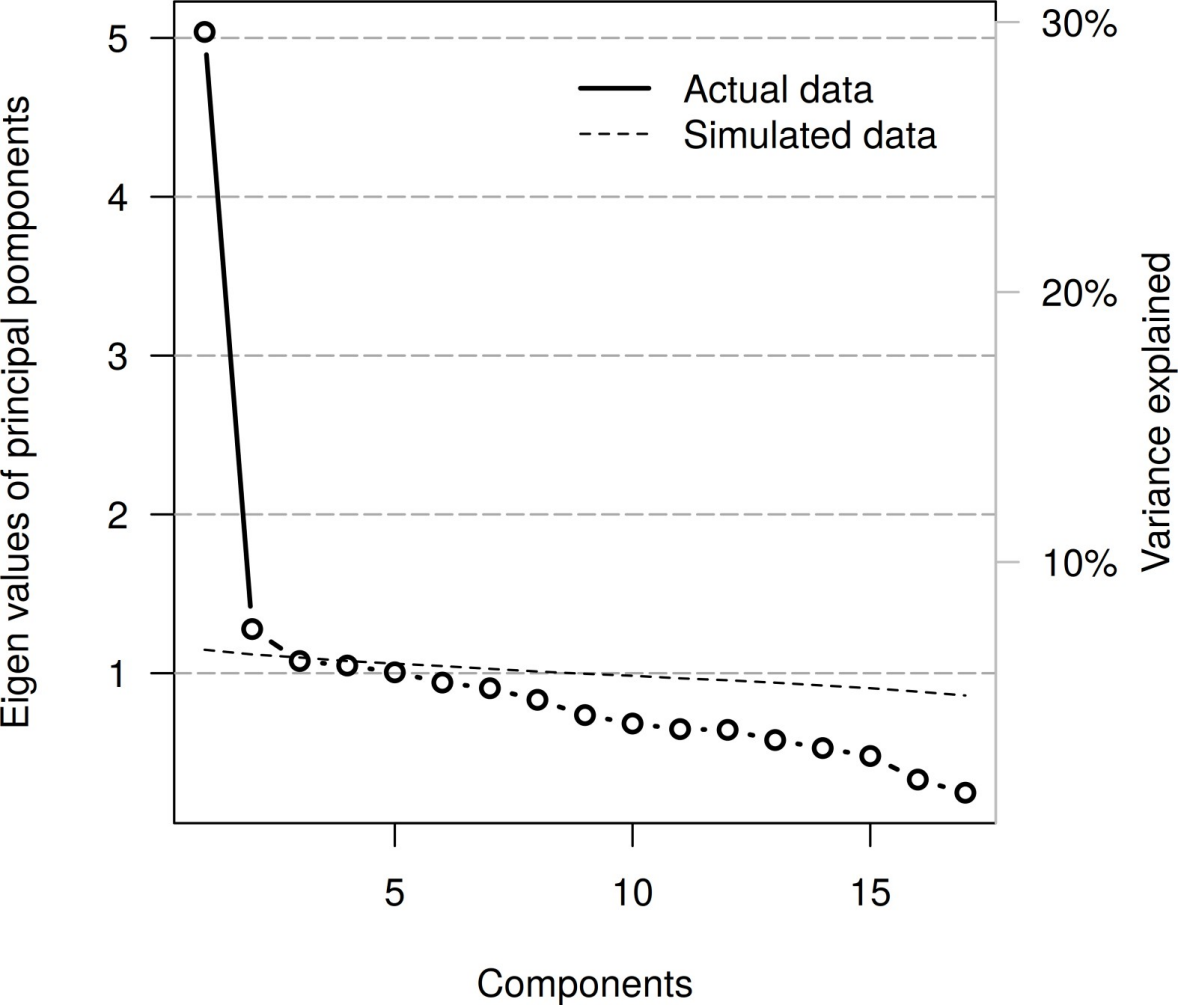
Scree plot indicating that one factor suffices to describe the variability across all items of the full neurocognitive test battery. The dashed line is a parallel analysis via Monte Carlo simulation. PC: principle components.

Fig 1 shows a parallel plot for the PCA on the 17 items. Clearly, the eigenvalue and explained variance of the first component is highest. The fact that the scree line lies slightly above the line of simulated eigenvalues at component two suggest that a two-factor model might be more in place than a one-factor model to account for the variance in all 17 items. Based on this suggestion, we estimated an exploratory 2-factor model, inspecting results after a varimax rotation.

Inspecting the factor loadings of the exploratory two-factor model revealed two undesirable results. First, the Trail Making Test that is part of the MoCA did not end up in the same factor as the two separately administered Trail Making Tests (TMT-A and TMT-B), which is difficult to explain. Second, results indicated a number of rather large cross loadings (first and fourth column of S2 Table). These medium-sized and rather “undecided” factor loadings prompted us to inspect the stability of the factor-item mappings. We did so by estimating the same exploratory two-factor model on three subsets of the data, each omitting one third of the data. Each of these subsets yielded rather different item loadings and, most crucially, the factor that each item was assigned to based on these factor loadings differed between subsets (factor loadings of the two-factor model are shown in S2 Table). We did nonetheless fit the two-factor confirmatory factor model (assigning each item to the factor where it had the highest loading in the EFA) and compared the model performance with the one-factor model. Here, we made use of the Bayesian Information Criterion (BIC), a measure of model performance that balances the complexity of the model in terms of the number of parameters against model fit, with lower BICs indicating better models [39, 40]. The model comparison favored the two-factor model, indicating a one-factor BIC of 107386.9 and a two-factor BIC of 107129.7. Note that this model comparison is hard to interpret, because it is biased: the model structure that is tested is based on the exploratory EFA, creating a real risk of overfitting [41].

This consideration, and the undesirable properties in terms of interpretation mentioned above, are real drawbacks of using the two-factor model; it does not seem sensible to work in future research projects with an unstable two-factor model with loadings that are hard to interpret, of which we do not know whether it actually overfits the data. Further, the fit of both the one-factor and the two-factor model is excellent. Therefore, although the parallel plot and the BIC comparison favor a two-factor model, we decided that the one-factor model is of more value.

### Confirmatory factor analysis

#### Model fit

To study the absolute fit of our one-factor model, we calculated the most often-used fit indices to compare the fit of structural equation models (SEM). A Chi-square test for the fit of the model had χ2 of 455.5 on 119 degrees of freedom, leading to p <0.001; however, this small p may be a result of the large sample size rather than lack of fit. Our model had a Comparative Fit Index (CFI) of 0.974. The CFI is an index that quantifies the relative fit of the fitted model compared to a null model, where all variables are uncorrelated. Typically, values of CFI > 0.9 are considered moderate, > 0.95 good. The Tucker-Lewis index (TLI), which is a measure of discrepancy between the chi-squared value of the fitted model and the chi-squared value of the null model and supposed to indicate good fit when >0.95, was found to be 0.97. We also calculated the Root Mean Square Error of Approximation (RMSEA) to be 0.034 for our model; RMSEA is typically considered good whenever < .05. Finally, we found Standardized Root Mean Square Residual (SRMR)–a goodness of fit index considered good when < 0.08 –of 0.052. When each of these measures of model performance indicate a good model fit, as it is the case for our models, appropriate fitting of the model can be assumed.

#### Measurement invariance over time

The one-factor model derived above will be used to calculate scores not only at baseline, where we fitted the model, but also on future measurements in the Swiss-AF cohort. Therefore, it is essential that the model measures at the same scale over time. Here, we assess this assumption of measurement invariance over time [42]. For this analysis, we included all observations from both the baseline measurement and the first follow-up measurement. The total number of patients with data for both baseline and follow-up is 2040. To this data set, we fitted two models. In the free model, each item’s factor loading was allowed to be different across the baseline and follow-up measurements. In the time-invariant model, each item’s factor loading was constrained to be equal for baseline and follow-up.

Table 4 shows the results of comparing the time-invariant model against the free model. This comparison is based on a model fit where ordinal items were treated as numeric items, allowing for calculating the ML estimates and deriving a likelihood ratio test and AIC and BIC model performance measures. As apparent from the table, the likelihood ratio test nominally prefers the time-invariant model. The improvement, however, is modest, and the BIC favors the time-invariant model by a considerable margin (ΔBIC = 81.47). Inspection of the factor loadings of the free model also revealed that all loadings were very similar across time points. Combined with the observation that the invariant model fits the data well (CFI = 0.978, RMSEA = 0.033, SRMR = 0.056, TLI = 0.975), we judged that the assumption of time-invariance is a defensible simplification for the factor score that we derived above.

**Table 4:** Comparison of the free model to a model where factor loadings were constrained to be time-invariant across baseline and follow-up.

	Df	AIC	BIC	Chisq	Chisq diff	Df diff	Pr(>Chisq)
Free	509	197215.8	197910.49	1971.86			
Time invariant	525	197226.95	197829.01	2015.01	36.87	16	0

#### Measurement invariance across groups

Since we aimed to compare different groups and types of AF patients using the factor score and anticipate that future LNCCIs and SNCIs possibly affect this score, we performed tests of measurement invariance across a selection of grouping variables groups, comparing 1) patients younger and older than median, 2) patient sex, 3) patient education (3 groups), 4) AF type, 5) presence of LNCCIs, and 6) presence of SNCIs. For several tests, the likelihood ratio test favored the time-variant model, but for all tests, the BIC favored the group-invariant model. Model comparisons were again based on a model fit where ordinal items were treated as numeric items.

Based on this, we believe it is a tenable simplification to assume measurement invariance across groups. Concluding, these tests of measurement invariance can be seen as reassuring the interpretation of the factor score in future Swiss-AF analyses.

### Sensitivity of the factor score

**Fig 2:**
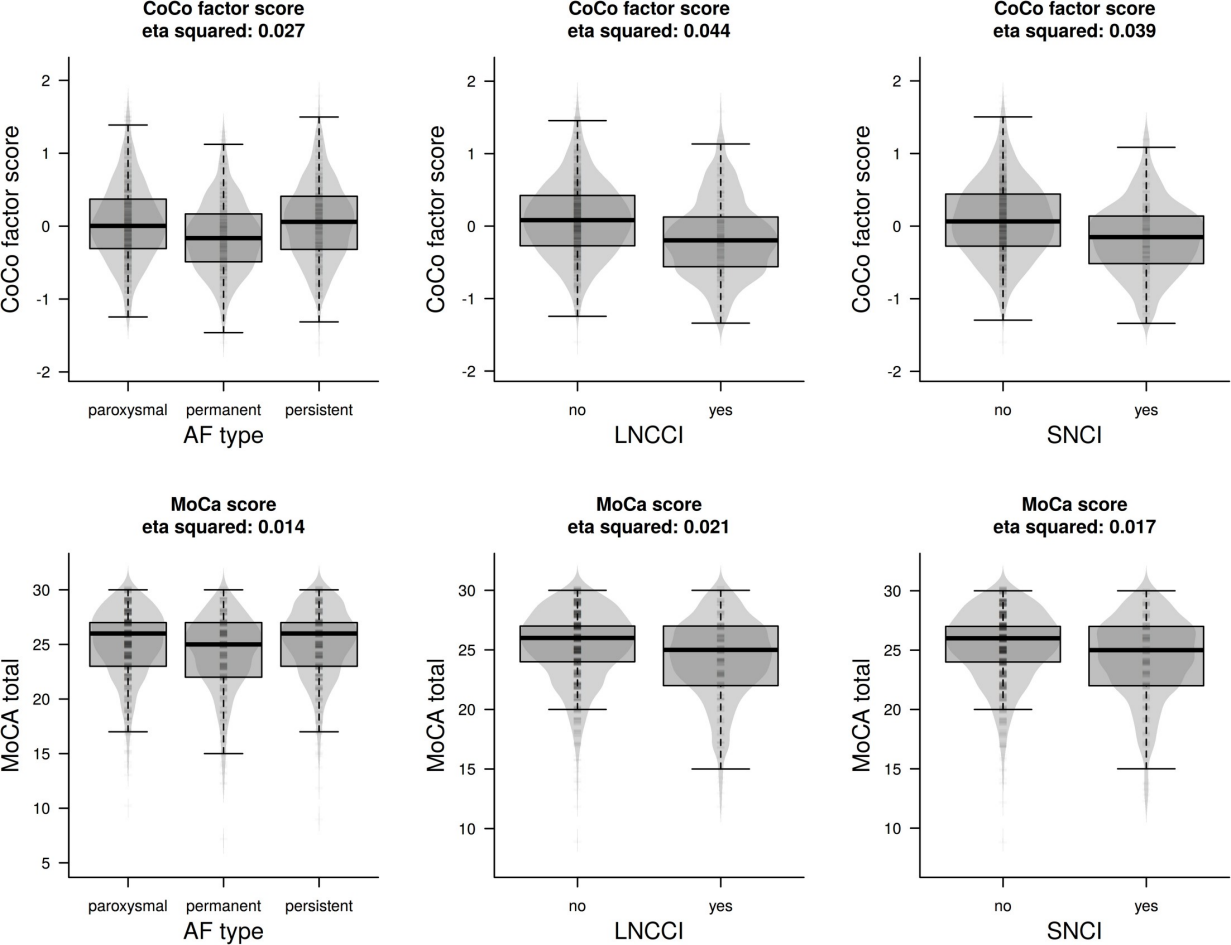
Distribution of the CoCo factor score (upper row of panels) and MoCA score (lower row of panels) stratified by AF type, LNCCI, and SNCI. On the background of each box, a density plot of the underlying data is shown in light grey, as well as lines indicating the individual data points (with some jitter). The figure titles indicate the eta^2^, which is representing the observed effect size. These eta^2^ are based on ANOVA’s. Specifically, eta^2^ is the grouping sums of squares divided by the total sum of squares (further explanations in the text).

To study whether the CoCo score indeed offers a more sensitive measure of cognitive impairment than the MoCA score alone, we investigated how strongly both scores relate to a number of variables that are expected to be associated with cognitive decline. Fig 2 shows the distribution of CoCo factor scores (upper row of panels) and MoCA scores (lower row of panels) as a function of three such covariates: AF type, the presence of LNCCIs, and the presence of SNCIs. In each figure title, effect size between levels of the covariate is indicated as eta^2^. For AF types, LNCCIs, and SNCIs, the obtained effect sizes are clearly larger for the CoCo score than for the MoCA score, reaching a factor of two in case of LNCCIs and SNCIs.

**Fig 3:**
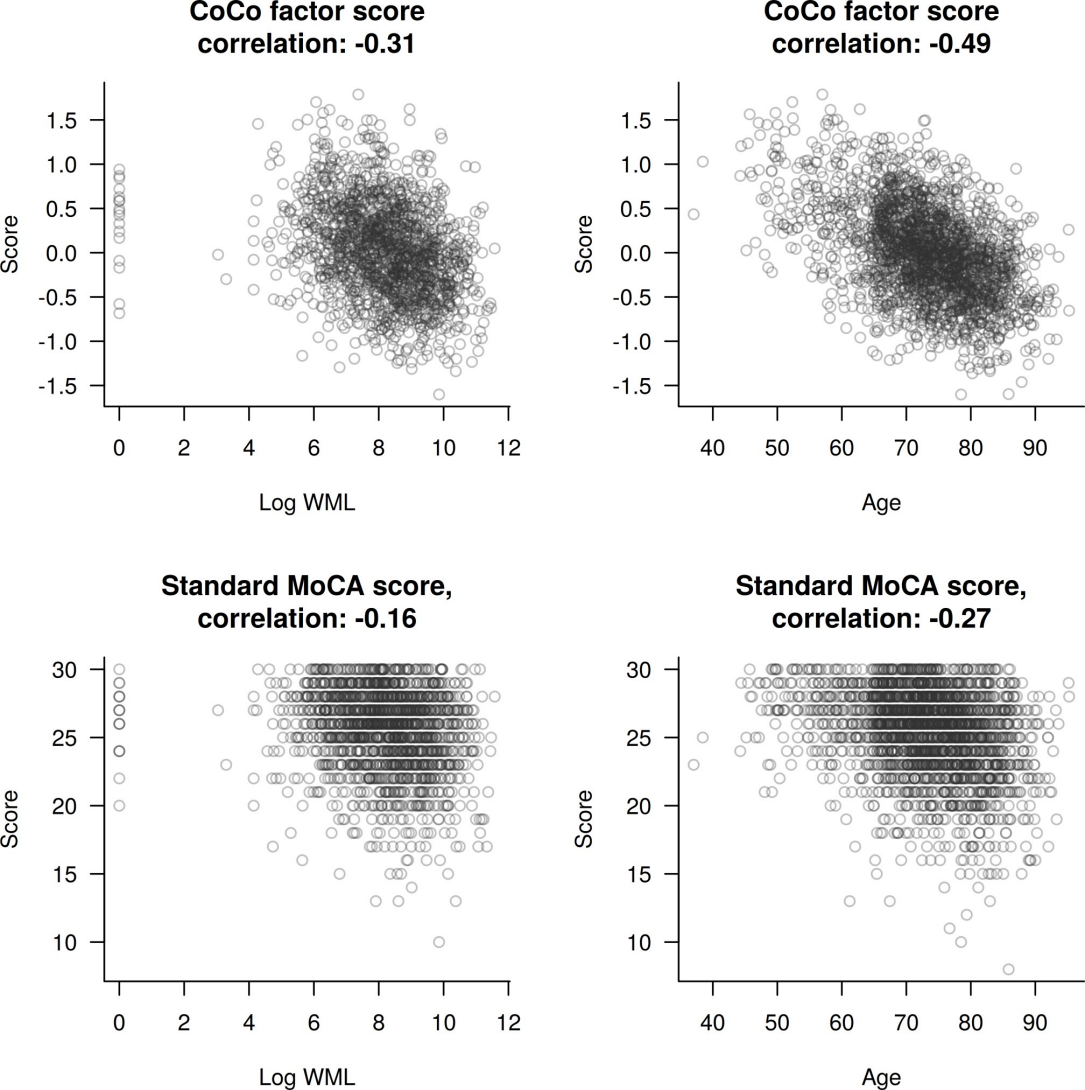
Factor scores (odd rows) and MoCA sum scores (even rows, corrected for education) as a function of relevant covariates (WML; age). Titles show the observed correlations.

Fig 3 shows both the CoCo score and the MoCA score as a function of two continuous variables: age and the size of observed WML. Again, the effect size, here in terms of the correlation, are clearly larger for the CoCo score, suggesting that the CoCo score allows for more fine-grained analysis of MCI in AF patients relative to existing neurocognitive measures.

## Discussion

Swiss-AF is a large prospective cohort study of mainly elderly patients with AF. It provides a contemporary interdisciplinary platform to study the interrelationships of AF, brain lesions and cognitive impairment from a longitudinal perspective. Furthermore, it illuminates the impact of AF progression on the quality of life, symptoms, and cardiovascular and non-cardiovascular outcomes.

The present analysis constructed a factor score–the CoCo (cognitive construct) score–which can be used as a summary measure of the common aspects of all items obtained from four validated neurocognitive assessments included in the Swiss-AF study. This CoCo score revealed good psychometric properties and appropriately accounted for a relevant amount of variance in all items from the full neurocognitive test battery used in this study. Results showed that measurement invariance was present over time and across a number of relevant grouping variables, which is essential for further analyses, allowing for a more sensitive measurement of longitudinal cognitive performance in patients of the Swiss-AF cohort. Correspondingly, we will use the CoCo score in future studies to investigate associations with other key factors determining the development of MCI in AF patients, including brain lesions, comorbidities, medication, and lifestyle (like alcohol consumption, smoking, physical activity).

### Study strengths and limitations

This study includes a large number of well-characterized AF patients, recruited from the main language regions of Switzerland, with few missing values. Hence, the results can be taken to represent the study population very well.

The CoCo score derived in this study reflects our best possible model for the set of baseline data at hand, capturing the common aspects of the different cognitive functions measured by the neurocognitive tests used in the Swiss-AF cohort. Hence, it provides a summary of the items included in our neurocognitive test battery that is more sensitive than the previously used main indicator of cognition in the Swiss-AF cohort study, the MoCA score [6].

Specifically, the sensitivity of the MOCA score is compromised by a ceiling effect, with 30 being the maximal attainable score. This is not the case with the CoCo score, making it more sensitive relative to the MoCA score. This advantage of the new CoCo score is also illustrated by Figs 2 and 3 showing the distribution of the MoCA and the CoCo scores according to AF type, LNCCI, and SNCI as crucial risk factors (Fig 2) and as a function of age and WML as relevant covariates (Fig 3). Here, the differences indicated for CoCo scores are larger than those indicated for MoCA scores.

Using the CoCo score allows for a one-dimensional interpretation of results, taking advantage of all the information about the patients’ cognitive abilities that is tapped by the items from the different neuropsychological tests in the test battery. By combining all shared variance that the constituent items have in common, the CoCo score offers more power to detect relationships with other variables. Put differently, by avoiding the number of statistical tests needed when analyzing each of the neuropsychological test scores separately, the CoCo score allows for more robust and fine-grained analyses of the associations between cognition and a large number of key risk factors in AF patients, including brain lesions, clinical variables, medication, and lifestyle, corresponding to the main research aims of the Swiss-AF cohort.

This advantage of using only one score indicating cognitive functioning will be valuable especially for the conduct of longitudinal investigations of neurocognitive performance changes in AF patients over time (e.g., 10 years after baseline).

The fact that we were able to show that the CoCo score shows a relatively strong association with key covariates such as WML and the presence of LNCCIs suggests that this battery of items captures a facet of neurocognitive abilities that is highly relevant for AF populations. This finding can also serve as a starting point to explore new ways of measuring MCI that go beyond the use of the established MoCA score.

Correspondingly, although beyond the scope of the present study, a remark should be made with regard to the developed cognitive construct. Our results confirm that measurement properties can be optimized by forming a cognitive factor score including items beyond the item set of the MoCA test. Thus, a stronger focus on psychomotor speed and mental flexibility (as was robustly captured by TMT, SFT and DSST) may enrich our understanding of cognitive impairment in AF patients, beyond the cognitive domains assessed by the MoCA test. It is up to future studies to evaluate the predictive and discriminatory potential of the proposed cognitive item set with respect to screening and modeling the development of MCI and dementia in patients with AF.

Despite the above, the following limitations of the present study should be considered. First, the vast majority (87%) of patients included is over 65 years old (n = 2,100), and virtually all patients (99.1%) are Caucasians (n = 2,394). Thus, the study population may not be representative of the full population of AF patients in Switzerland.

Furthermore, the interpretation of the proposed CoCo score is only possible in light of the items that were chosen as part of the neurocognitive test battery. For instance, as the test battery did not consider episodic memory, inhibition and planning, our score does not represent these functions. Extending the present findings to further cognitive functions in AF patients remains a promising future task.

Related to this point, the factor score derived in this study does not allow drawing conclusions about cognitive profiles that may include one ability more than others. Likewise, it does not allow to differentiate between the cognitive processes involved. To this aim, further studies are needed in which the corresponding validated tests used in the Swiss-AF cohort are considered.

Finally, we would like to point out that the factor score proposed here is not applicable as a clinically valid indication of MCI. The broader Swiss-AF project, of which this factor score is a building block, may deepen our understanding of AF-related MCI such that it is eventually possible to formulate a formal criterion of MCI.

The factor score developed in this study was designed as a tool for AF researchers studying the relationships between cognition and a large number of key risk factors including disability, medication adherence and lifestyle. Results of these studies will improve our understanding of AF and possible treatment abilities.

## Supporting information

S1 File Swiss-AF investigators. (DOCX)

S1 Table Correlations among all items from all neurocognitive tests, the MoCA score, and the CoCo score. (DOCX)

S2 Table Item loadings in exploratory factor analysis with two factors (F1 and F2). Sets 1, 2, and 3 are various non-exclusive subsamples of the data. See main text for details. In bold is, for each set and item, the highest loading, assigning an item to either factor 1 or 2.
(DOCX)
