# Supplementary material for: A factor score reflecting cognitive functioning in patients from the Swiss Atrial Fibrillation Cohort Study (Swiss-AF)
Source: PLoS One. 2020 Oct 9;15(10):e0240167. doi: 10.1371/journal.pone.0240167 (PMC7546506; doi:10.1371/journal.pone.0240167)
Supplement: S1 File — (DOCX) [file pone.0240167.s001.docx]

**University Hospital Basel and Basel University:** Stefanie Aeschbacher, Chloé Auberson, Steffen Blum, Leo Bonati, Selinda Ceylan, David Conen, Simone Doerpfeld, Ceylan Eken, Marc Girod, Elisa Hennings, Philipp Krisai, Michael Kühne, Christine Meyer-Zürn, Pascal Meyre, Andreas U. Monsch, Christian Müller, Stefan Osswald, Anne Springer, Christian Sticherling, Thomas Szucs, Gian Voellmin, Leon Zwimpfer. Local Principal Investigator: Michael Kühne; Principal Investigator: Stefan Osswald (Email: [stefan.osswald@usb.ch](mailto:stefan.osswald@usb.ch))

**University Hospital Bern:** Faculty: Drahomir Aujesky, Urs Fischer, Juerg Fuhrer, Laurent Roten, Simon Jung, Heinrich Mattle; Research fellows: Luise Adam, Carole Elodie Aubert, Martin Feller, Axel Loewe, Elisavet Moutzouri, Claudio Schneider; Study nurses: Tanja Flückiger, Cindy Groen, Lukas Ehrsam, Sven Hellrigl, Alexandra Nuoffer, Damiana Rakovic, Nathalie Schwab, Rylana Wenger. Local Principal Investigator: Nicolas Rodondi (Email: [Nicolas.Rodondi@insel.ch](mailto:Nicolas.Rodondi@insel.ch))

**Stadtspital Triemli Zurich:** Christopher Beynon, Roger Dillier, Michèle Deubelbeiss, Franz Eberli, Christine Franzini, Isabel Juchli, Claudia Liedtke, Jacqueline Nadler, Thayze Obst, Jasmin Roth, Fiona Schlomowitsch, Xiaoye Schneider, Katrin Studerus, Noreen Tynan, Dominik Weishaupt. Local Principal Investigator: Andreas Müller (Email: [AndreasStephan.Mueller@triemli.zuerich.ch](mailto:AndreasStephan.Mueller@triemli.zuerich.ch))

**Kantonspital Baden:** Simone Fontana, Silke Kuest, Karin Scheuch, Denise Hischier, Nicole Bonetti, Alexandra Grau, Jonas Villinger, Eva Laube, Philipp Baumgartner, Mark Filipovic, Marcel Frick, Giulia Montrasio, Stefanie Leuenberger, Franziska Rutz. Local Principal Investigator: Jürg-Hans Beer (Email: [hansjuerg.beer@ksb.ch](mailto:hansjuerg.beer@ksb.ch))

**Cardiocentro Lugano:** Angelo Auricchio (lead author, Email: [Angelo.Auricchio@cardiocentro.org](mailto:Angelo.Auricchio@cardiocentro.org)), Adriana Anesini, Cristina Camporini, Giulio Conte, Maria Luce Caputo, Francois Regoli. Local Principal Investigator: Tiziano Moccetti

**Kantonsspital St. Gallen:** Roman Brenner, David Altmann, Michaela Gemperle. Local Principal Investigator: Peter Ammann (Email: [peter.ammann@kssg.ch](mailto:peter.ammann@kssg.ch))

**Hôpital Cantonal Fribourg:** Mathieu Firmann, Sandrine Foucras, Martine Rime. Local Principal Investigator: Daniel Hayoz (Email: [Daniel.Hayoz@h-fr.ch](mailto:Daniel.Hayoz@h-fr.ch))

**Luzerner Kantonsspital:** Benjamin Berte, Virgina Justi, Frauke Kellner-Weldon, Brigitta Mehmann, Sonja Meier, Myriam Roth, Andrea Ruckli-Kaeppeli, Ian Russi, Kai Schmidt, Mabelle Young. Local Principal Investigator: Richard Kobza (Email: [richard.kobza@luks.ch](mailto:richard.kobza@luks.ch))

**Ente Ospedaliero Cantonale Lugano:** Luisa Vicari, Jane Frangi-Kultalahti, Tatiana Terrot. Local Principal Investigator: Giorgio Moschovitis (Email: [Giorgio.Moschovitis@eoc.ch](mailto:Giorgio.Moschovitis@eoc.ch))

**University Hospital Geneva:** Georg Ehret, Hervé Gallet, Elise Guillermet, Francois Lazeyras, Karl-Olof Lovblad, Patrick Perret, Philippe Tavel, Cheryl Teres. Local Principal Investigator: Dipen Shah (Email: [Dipen.Shah@hcuge.ch](mailto:Dipen.Shah@hcuge.ch))

**University Hospital Lausanne:** Nathalie Lauriers, Marie Méan, Sandrine Salzmann. Local Principal Investigator: Jürg Schläpfer (Email: [Jurg.Schlaepfer@chuv.ch](mailto:Jurg.Schlaepfer@chuv.ch))

**Bürgerspital Solothurn:** Jan Novak, Andrea Grêt, Pia Schnyder, Sandra Vitelli. Local Principal Investigator: Frank-Peter Stephan (Email: [frank-peter.stephan@spital.so.ch](mailto:frank-peter.stephan@spital.so.ch))

**Ente Ospedaliero Cantonale Bellinzona:** Jane Frangi-Kultalahti, Augusto Gallino. Local Principal Investigator: Marcello Di Valentino (Email: [Marcello.DiValentino@eoc.ch](mailto:Marcello.DiValentino@eoc.ch))

**University of Zurich / University Hospital Zurich:** Fabienne Witassek, Matthias Schwenkglenks (Email: [matthias.schwenkglenks@uzh.ch](mailto:matthias.schwenkglenks@uzh.ch))

**Medical Image Analysis Center AG Basel:** Jens Würfel (Head, Email: [jw@miac.ch](mailto:jw@miac.ch)), Anna Altermatt, Michael Amann, Petra Huber, Esther Ruberte, Tim Sinnecker, Vanessa Zuber

**Clinical Trial Unit, University Hospital Basel:** Michael Coslovsky (Head, Email: [michael.coslovsky@usb.ch](mailto:michael.coslovsky@usb.ch)), Pascal Benkert, Gilles Dutilh, Andrea Wiencierz, Milica Markovic, Pia Neuschwander, Patrick Simon

**Schiller AG Baar:** Ramun Schmid (Email: [Ramun.Schmid@schiller.ch](mailto:Ramun.Schmid@schiller.ch)).
