# Supplementary figures and images for: A factor score reflecting cognitive functioning in patients from the Swiss Atrial Fibrillation Cohort Study (Swiss-AF)
Source: PLoS One. 2020 Oct 9;15(10):e0240167. doi: 10.1371/journal.pone.0240167 (PMC7546506; doi:10.1371/journal.pone.0240167)

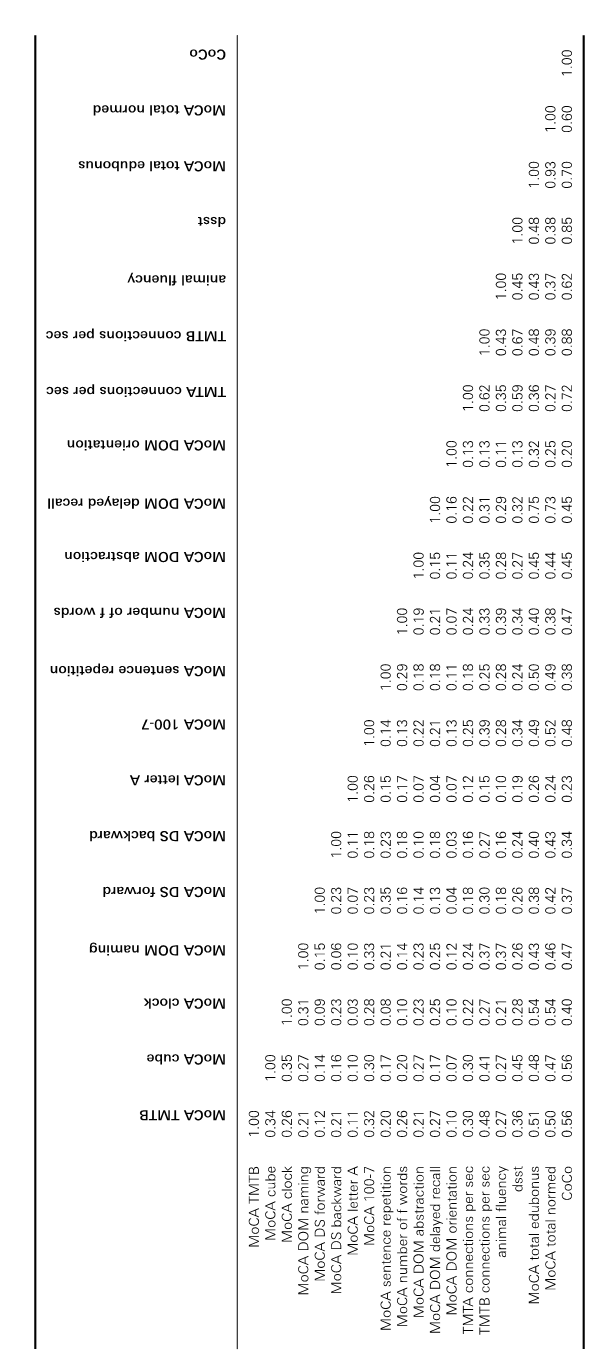

Supplement: S1 Table — (DOCX) [file pone.0240167.s002.docx]
