# Supplementary material for: A factor score reflecting cognitive functioning in patients from the Swiss Atrial Fibrillation Cohort Study (Swiss-AF)
Source: PLoS One. 2020 Oct 9;15(10):e0240167. doi: 10.1371/journal.pone.0240167 (PMC7546506; doi:10.1371/journal.pone.0240167)
Supplement: S2 Table — Sets 1, 2, and 3 are various non-exclusive subsamples of the data. See main text for details. In bold is, for each set and item, the highest loading, assigning an item to either factor 1 or 2. (DOCX) [file pone.0240167.s003.docx]

|  | F1 all | F1 set 1 | F1 set 2 | F1 set 3 | F2 all | F2 set 1 | F2 set 2 | F2 set 3 |
| --- | --- | --- | --- | --- | --- | --- | --- | --- |
| MoCA TMT-B | 0.364 | 0.391 | **0.413** | 0.313 | **0.313** | **0.313** | 0.379 | **0.379** |
| MoCA Cube | 0.318 | 0.315 | 0.302 | **0.374** | **0.374** | **0.374** | **0.374** | 0.369 |
| MoCA Clock Drawing | 0.123 | 0.148 | 0.132 | 0.126 | **0.126** | **0.126** | **0.126** | **0.126** |
| MoCA Naming Animals | 0.189 | 0.12 | 0.23 | 0.241 | **0.241** | **0.241** | **0.241** | **0.241** |
| MoCA Digit Span forward | 0.223 | 0.213 | **0.315** | 0.206 | **0.206** | **0.206** | 0.133 | **0.133** |
| MoCA Digit Span backward | 0.188 | 0.199 | 0.248 | 0.158 | **0.158** | **0.158** | **0.158** | **0.158** |
| MoCA Letter A | 0.134 | **0.181** | 0.079 | 0.181 | **0.181** | 0.175 | **0.175** | **0.175** |
| MoCA 100-7 | 0.227 | 0.266 | 0.302 | 0.167 | **0.167** | **0.167** | **0.167** | **0.167** |
| MoCA Sentence Repetition | 0.185 | 0.174 | **0.294** | 0.155 | **0.155** | **0.155** | 0.219 | **0.219** |
| MoCA Number of F-words | 0.295 | 0.288 | **0.402** | 0.259 | **0.259** | **0.259** | 0.253 | **0.253** |
| MoCA Abstraction | 0.25 | 0.281 | 0.234 | 0.289 | **0.289** | **0.289** | **0.289** | **0.289** |
| MoCA Delayed Recall | 0.227 | 0.229 | 0.263 | 0.245 | **0.245** | **0.245** | **0.245** | **0.245** |
| MoCA Orientation | 0.068 | 0.085 | 0.074 | 0.074 | **0.074** | **0.074** | **0.074** | **0.074** |
| TMT-A, connections per sec | **0.763** | **0.772** | **0.741** | **0.799** | 0.183 | 0.149 | 0.156 | 0.163 |
| TMT-B, connections per sec | **0.761** | **0.771** | **0.814** | **0.737** | 0.428 | 0.389 | 0.349 | 0.469 |
| Semantic Fluency, Animals | 0.374 | 0.37 | 0.424 | 0.406 | **0.406** | **0.406** | **0.406** | **0.406** |
| DSST | **0.721** | **0.713** | **0.742** | **0.726** | 0.375 | 0.377 | 0.327 | 0.364 |
